# An Efficient Immunization Strategy for Community Networks

**DOI:** 10.1371/journal.pone.0083489

**Published:** 2013-12-20

**Authors:** Kai Gong, Ming Tang, Pak Ming Hui, Hai Feng Zhang, Do Younghae, Ying-Cheng Lai

**Affiliations:** 1 Web Sciences Center, University of Electronic Science and Technology of China, Chengdu, People's Republic of China; 2 Department of Physics, The Chinese University of Hong Kong, Shatin, Hong Kong, People's Republic of China; 3 Department of Mathematics, Kyungpook National University, Daegu, South Korea; 4 School of Mathematical Science, Anhui University, Hefei, People's Republic of China; 5 School of Electrical, Computer and Energy Engineering, Arizona State University, Tempe, Arizona, United States of Ameica; Hungarian Academy of Sciences, Hungary

## Abstract

An efficient algorithm that can properly identify the targets to immunize or quarantine for preventing an epidemic in a population without knowing the global structural information is of obvious importance. Typically, a population is characterized by its community structure and the heterogeneity in the weak ties among nodes bridging over communities. We propose and study an effective algorithm that searches for bridge hubs, which are bridge nodes with a larger number of weak ties, as immunizing targets based on the idea of referencing to an expanding friendship circle as a self-avoiding walk proceeds. Applying the algorithm to simulated networks and empirical networks constructed from social network data of five US universities, we show that the algorithm is more effective than other existing local algorithms for a given immunization coverage, with a reduced final epidemic ratio, lower peak prevalence and fewer nodes that need to be visited before identifying the target nodes. The effectiveness stems from the breaking up of community networks by successful searches on target nodes with more weak ties. The effectiveness remains robust even when errors exist in the structure of the networks.

## Introduction

Epidemics could lead to a serious loss in life and have a huge impact on the economy, as we witnessed during the outbreaks of SARS (severe acute respiratory syndromes) in 2003 and H1N1 Influenza A virus in 2009 [1]–[3]. The problem of preventing an epidemic in time is of great importance and it has attracted much attention from researchers across different fields [4]–[12]. While immunization and quarantine are the two basic measures [13]–[15], finding an efficient way of identifying the targets to be immunized or quarantined so as to suppress an infection effectively remains a pressing issue [16].

The hubs, individuals with high centrality, in complex networks are commonly believed to be the most influential nodes as they could affect their many neighboring nodes [17]. Kitsak *et al.* pointed out that the nodes with a high 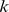-shell are more influential spreaders in some real networks [18]. Undoubtedly, it is important to identify such influential nodes, but an effective method would require *global* information about the network structure. In these global strategies, the influential hubs can be identified by centrality measures, such as the degree centrality [19], [20], eigenvector centrality [21], and betweenness centrality [22], [23]. Such global strategies, however, become impractical for large-scale networks. Another type of method requires only *local* information. The acquaintance immunization strategy (henceforth labeled as ACQ) [24] in which random acquaintances of randomly chosen nodes are immunized is an example of local search algorithms. ACQ was shown to be an efficient algorithm for large-scale networks with broad-degree distributions [24].

*Community structure* at the mesoscale level is ubiquitous in a variety of real complex systems [25], [26] such as Facebook [27], [28] and Twitter [29], which plays an important role in the dynamics of epidemics [30]–[35]. In the presence of communities, the weak ties connecting a pair of nodes belonging to different communities, called the *bridge nodes* [36]–[38], provide the pathways for information and diseases to propagate from one community to another. These bridge nodes were found to be more important than the hubs in diffusing information through community networks [39]–[41]. Therefore, identifying the bridge nodes in community networks are crucial in preventing epidemic outbreaks [42]–[44]. Deterministic and stochastic algorithms have been designed to search for the bridge nodes [45]. Chen *et al.* [46] proposed an immunization strategy based on an equal graph partitioning algorithm to identify the minimum group that separates a network into several clusters of approximately equal size. The role of the minimum separator group is similar to that of the bridge nodes connecting different communities. The algorithm is effective in that only a small immunization ratio is needed, which is the fraction of immunized nodes. However, the algorithm requires the structural information of the whole network. Similar to other global strategies, it becomes impractical due to the high computing cost and, more fundamentally, the lack the complete information in large networks. Salathe and Jones [45] proposed the *community bridge finder* (CBF), which is a stochastic algorithm to search for the bridge nodes based only on local structural information, and found that the method is more efficient than immunization strategies targeting different kinds of hubs.

Community networks typically exhibit a heterogeneous distribution in the number of weak ties originating from a bridge node [36], [47]. In the worldwide air traffic network, for example, bridge nodes can be divided into four classes: nonhub connector nodes, nonhub kinless nodes, connector hubs, and kinless hubs [48]. It was found that immunizing bridge hubs that connect a community to many other communities could protect a network efficiently against epidemics [49]. Identifying the bridge hubs with a larger number of weak ties, however, is quite challenging as the mesoscopic-scale community structure is difficult to resolve for large-scale networks [50]. It is against this backdrop that we propose an immunization strategy that identifies the bridge hubs by random walks in an expanding friendship circle. The strategy, which we call bright-hub detector (BHD) relies only on local structural information. We compare results obtained by BHD with ACQ and CBF strategies and find that it performs better in simulated networks and empirical community networks generated from real data. Simulation results also show that our strategy has the merit of being robust against noise.

## Results

**Figure 1:**
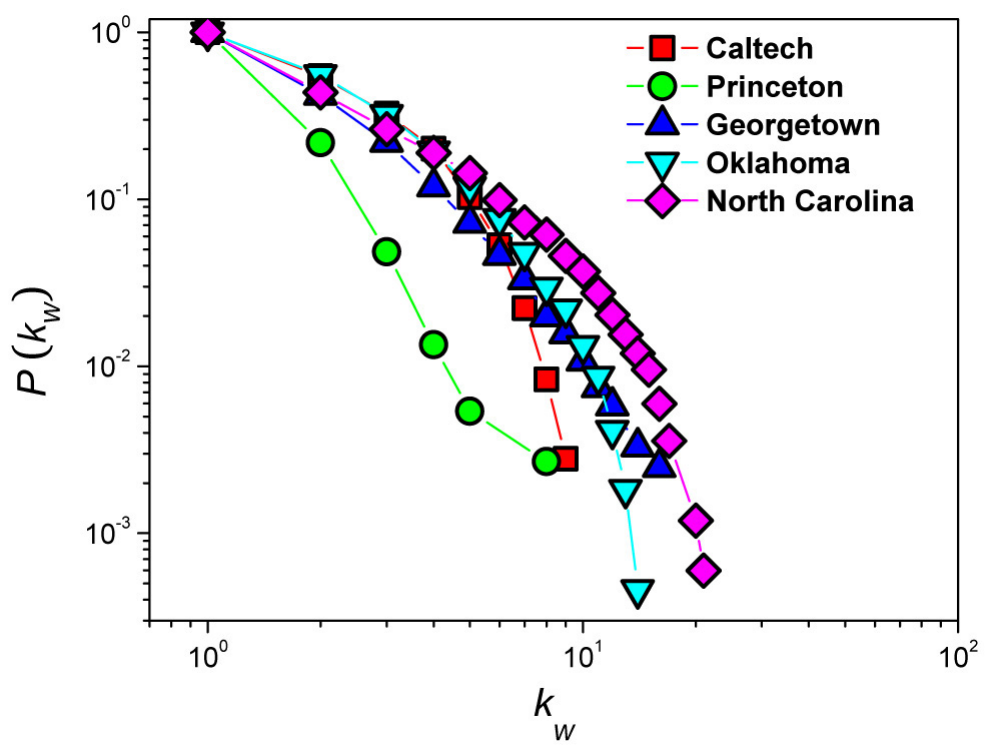
Cumulative distributions of the number of weak ties in five empirical networks. For every network, the community structure is detected by the method proposed by Newman and Girvan [55]. Weak ties and bridge nodes are then identified. The number of weak ties emanating from each bridge node is recorded to give the cumulative distribution. Results are shown for students' networks for Caltech (red squares), Princeton (green circles), Georgetown (blue up triangles), Oklahoma (cyan down triangles) and North Carolina (magenta diamonds).

To illustrate the community structure in real-world networks and the necessity of an algorithm that focuses on identifying the bridge hubs, we have analyzed the heterogeneity of bridge nodes in the networks of students in five universities in the US using Facebook data. Details on constructing the networks from data are given in the Materials and Methods section. Figure 1 shows the cumulative probability density 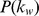 that a bridge node has a degree 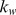 or higher in the students' Facebook networks of Caltech, Princeton, Georgetown, Oklahoma, and North Carolina. The distributions indicate that it is important to identify the bridge nodes with more weak ties, in addition to identifying only the bridge nodes as in CBF.

We present results of three different strategies (ACQ, CBF and BHD) on choosing immunizing nodes in simulated and empirical community networks within the susceptible-infected-recovered (SIR) epidemiological model. Briefly, simulated networks of different modularity 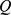 are generated by randomly connecting communities, which are themselves random networks, following the algorithm in Ref. [45]. Empirical networks are constructed from Facebook data of students in each of five US universities, with a link defined by two students being online friends and belonging to the same dormitory or the same major in the same year of study. Table 1 lists the properties of these empirical networks. Details about network construction, the three strategies, and SIR dynamics are given in the Materials and Methods section. The ACQ, CBF and BHD strategies rely only on local structural information. In ACQ [24], a node is randomly picked and then a neighbor of the chosen node is randomly selected for immunization. The algorithm has a higher chance to immunize the hubs. In CBF [45], a step forward in a self-avoiding walk is checked for a bridge between communities by examining the existence of links or paths from the neighbors of the last node back to the nodes visited in the trail of the self-avoiding walk. The node before crossing the bridge is then identified as the bridge node and immunized. In addition, the supplementary rule of immunizing a node that has been visited twice by self-avoiding walks would pick up some hubs. Here, we implemented CBF as given in Ref. [45]. The bridge-hub detector (BHD) that we proposed in this paper *extends* the self-avoiding searching scheme to examining the overlap and the existence of links from all the neighbors of the last node back to the *union of the friendship circles* of all the nodes in the trail of the walk. A pair of nodes, a bridge node and a bridge hub, are searched for immunization via a self-avoiding walk (Readers are referred to the Materials and Methods section for more information). In the SIR model, the parameters 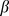 and 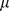 are the transmission rate and the recovery probability, respectively. The extent of an epidemic is characterized by the final epidemic ratio 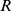, the fraction of the population ever infected at the end of the epidemic, and the peak prevalence 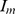, the highest density of infected nodes in the population at a time during the epidemic.

**Table 1:** Structural Properties of the five empirical networks.

Network	N	E	M	Q	r	H	C	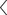 d 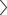	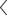 k 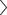
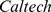	647	7,047	13	0.675	0.187	6.517	0.443	3.166	21.784
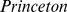	5,580	156,935	8	0.817	0.039	6.366	0.298	4.316	56.252
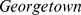	8,183	245,510	42	0.814	0.106	6.280	0.268	4.218	60.005
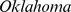	13,515	276,126	67	0.774	0.327	8.240	0.266	4.108	40.862
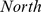 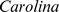	15,425	332,314	70	0.779	0.217	6.941	0.233	4.346	43.086

Structural properties including the network size (N), number of edges (E), the community number (M), modularity (Q) [55], degree assortativity (r) [56], degree heterogeneity (H = 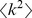/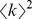)), clustering coefficient(C), average shortest path length (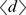) and average degree (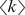) are tabulated for each of the five empirical networks.

**Figure 2:**
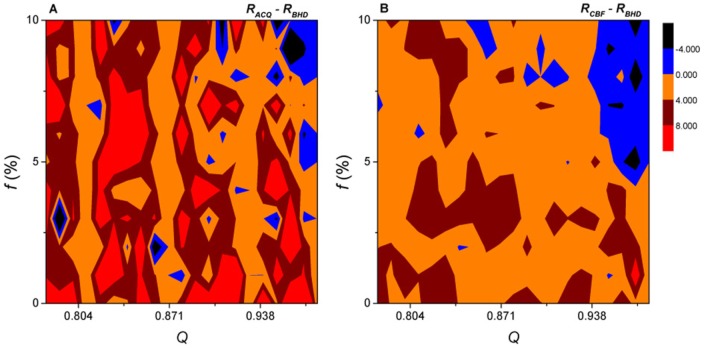
Comparison of efficacy of immunization algorithms in simulated networks. The difference in the final epidemic ratios (a) (left panel) 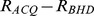 between ACQ and BHD, and (b) (right panel) 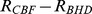 between CBF and BHD, are shown for simulated networks with different network modularity 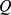 and immunization coverage 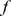. The colors indicate the differences in percentages (see color codes). Results are obtained by averaging over 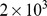 realizations for each pair of 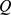 and 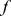 values. The parameters associated with the SIR dynamics are 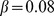 and 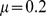.

To compare the performances of ACQ and CBF with that of BHD, we have carried out the SIR dynamics on simulated networks with different modularity. For a given immunization coverage 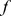, i.e., fraction of immunized nodes, the final epidemic ratio 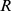 is obtained for each of the three strategies. The differences 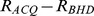 and 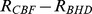 would indicate the performance of ACQ and CBF relative to BHD. Figure 2 shows the differences for various combinations of the modularity 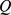 and coverage 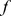. The differences are positive over most of the 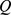-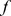 space, with BHD leading to about 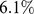 (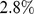) of fewer nodes being infected than ACQ (CBF). The results indicate that BHD is, in general, more effective in suppressing an epidemic. There are patches in the 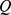-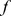 space that BHD is out-performed by ACQ and CBF. This is particularly apparent for networks with a strong community structure characterized by a high modularity. For example, both ACQ and CBF perform better in networks with 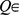
[0.95,0.97]. In these cases, an epidemic outbreak is mostly restricted in a local community and thus the better strategies are those that could confine and suppress an epidemic to within the community that the outbreak starts. Immunizing the hubs with large degrees as in ACQ and CBF can prevent epidemic spreading within a community more efficiently. BHD spends some of the immunization coverage on removing nodes that belong to neighboring communities rather than the community of the outbreak itself and this becomes less effective in saving the nodes within the outbreak community from infection than ACQ and CBF.

**Figure 3:**
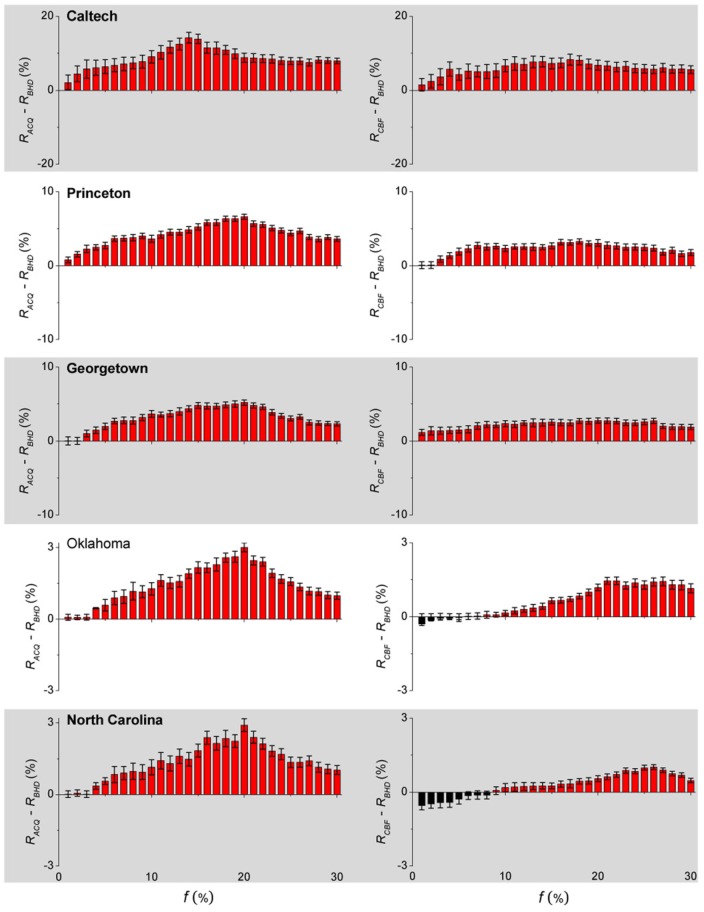
Comparison of efficacy of immunization algorithms in empirical networks. The differences in the final epidemic ratios 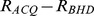 (left panel) and 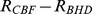 (right panel) using different immunization algorithms are shown for each of the five empirical networks as a function of the immunization coverage 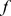. A positive value indicates that BHD is more effective than the other algorithms. Results are obtained by averaging over 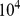 realizations for each value of 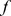.

Figure 3 shows the results of 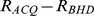 and 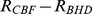 obtained for the empirical networks. In most cases, the final epidemic ratio is smaller for BHD than ACQ and CBF. For example, 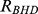 is on average 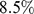 (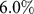) smaller than 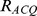 (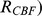 for the Caltech network, 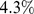 (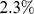) smaller for the Princeton network, and 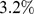 (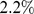) smaller for the Georgetown University network. For the Oklahoma University network, 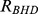 is about 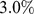 (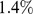) smaller than 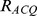 (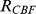) when the immunization coverage is not too small, e.g. 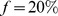 (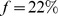). Similarly, 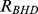 is about 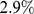 (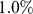) smaller than 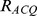 (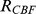) at 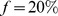 (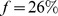) for the North Carolina University network. In the Oklahoma and North Carolina networks, BHD is out-performed by CBF when the immunization coverage is low. The ability of CBF to immunize the bridge nodes and some hubs makes it more effective in networks with a high assortativity and heterogeneity. As shown in Table 1, the degree assortativity 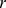 and heterogeneity 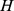 are 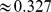 (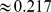) and 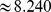 (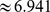) for the Oklahoma (North Carolina) University network. For an immunization coverage of 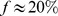, BHD performs better in all empirical networks than both ACQ and CBF, with a maximum in 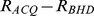 near this coverage. When the coverage is higher (e.g. 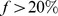), the differences in 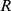 for the three algorithms diminish, as the many immunized nodes would tend to be effective in breaking up a community and suppress an epidemic regardless of the algorithm. The results for the differences in peak prevalence are given in Figure S1 and the features are essentially the same as those in Figure 3.

**Figure 4:**
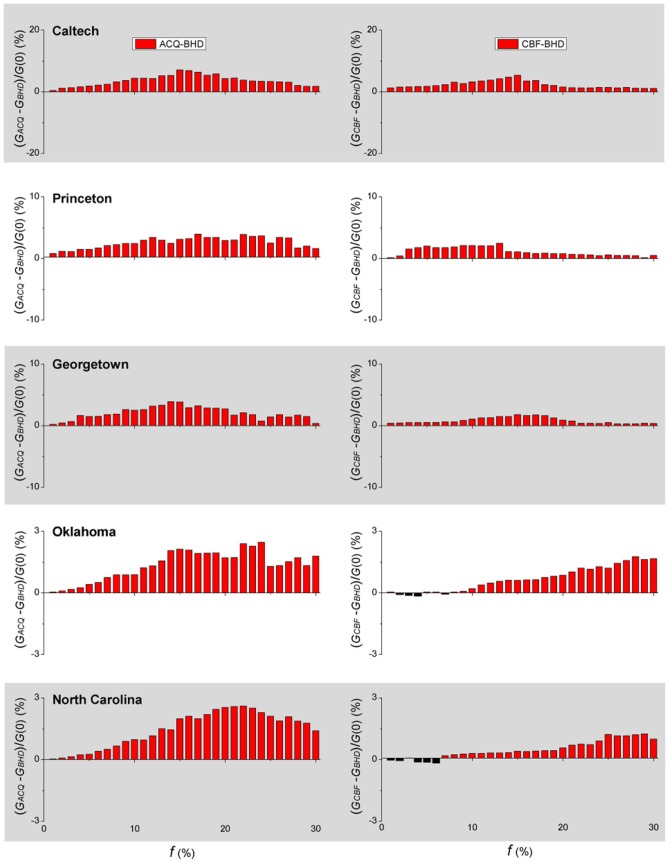
Comparison of giant components of different immunization algorithms in empirical networks. The difference in the sizes of the giant component 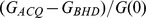 (left panel) and 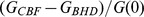 (right panel) using different immunization algorithms are shown for each of the five empirical networks as a function of the immunization coverage 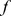, where 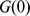 is the size of the giant component before an immunization algorithm is applied. A positive value indicates that BHD is more effective in breaking up the network. Results are obtained by averaging over 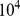 realizations for each value of 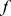.

Whether an algorithm is effective in preventing an epidemic is closely related to its effectiveness in breaking up the network after immunizing or removing a certain fraction of nodes [51]. Let 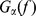, with 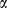 denoting ACQ, CBF and BHD, be the size of the giant component of the resulting network using the algorithm 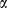 and an immunization coverage 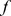, and 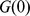 be the size of the giant component of the network before applying an immunization algorithm. The relative difference 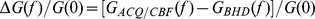 is a measure of how effective BHD breaks up a network relative to ACQ (and CBF). Figure 4 shows the results of 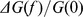 for the five empirical networks. The positive 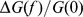 values in almost all the cases indicate that BHD is more effective. The results show a similar trend as a function of 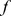 as in the differences in final epidemic ratio shown in Figure 3. Percentage wise, 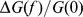 is smaller than 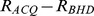 and 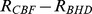 for the same values of 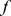 in the same empirical network. It is a result of the removal of some bridge hubs (bridge nodes with more weak links to other communities), by BHD, which would result in a network that has a smaller mean degree. This is indeed the case, as shown in Figure S2. The geometry of the resulting network also affects the duration of an epidemic. In particular, resulting networks with smaller mean degree lead to a longer duration, as the disease can only spread by infecting one node after another. BHD is therefore expected to have a longer epidemic duration, despite a smaller final epidemic ratio and prevalence. Figure S3 shows that the epidemic duration is slightly longer for BHD, as compared with those with ACQ and CBF.

**Figure 5:**
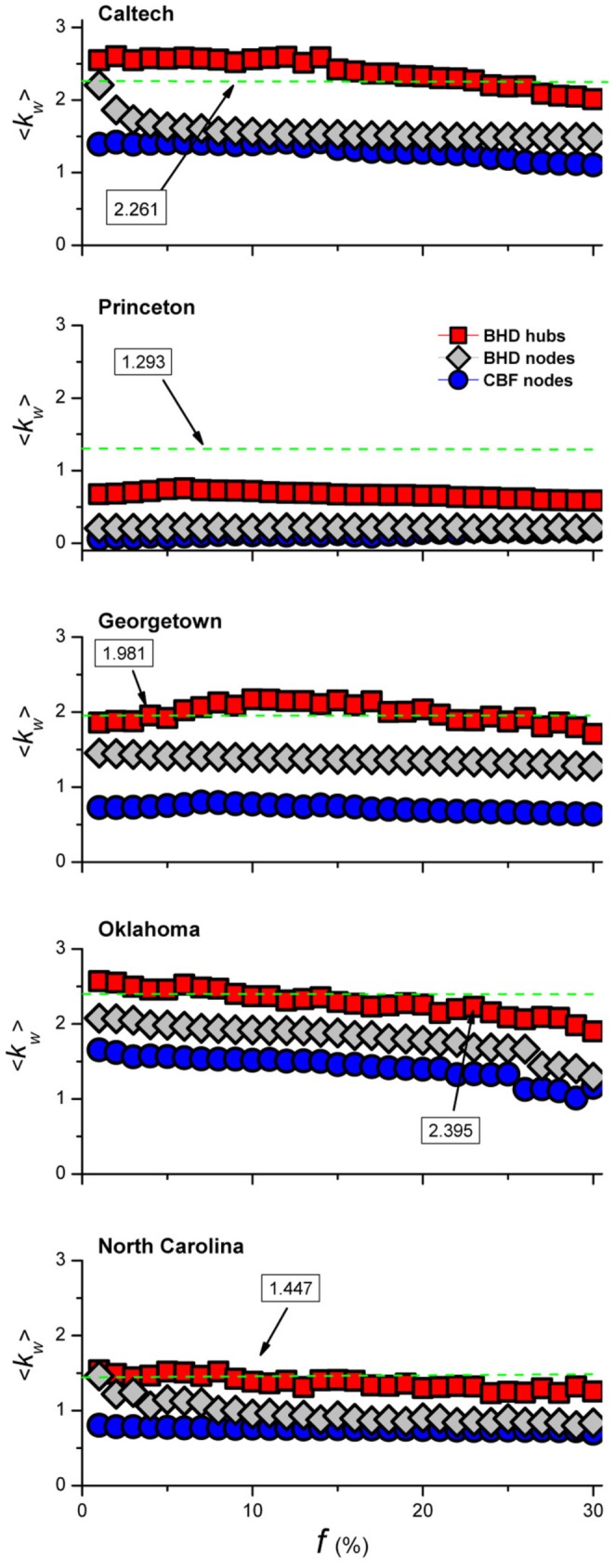
Comparison of average number of weak ties among immunized bridge hubs identified by BHD, bridge nodes identified by BHD and bridge nodes identified by CBF. BHD identifies a pair of nodes for immunization via a self-avoiding walk algorithm. One node is a bridge hub and another a bridge node. The average number of weak ties among these two types of immunized nodes [red squares (BHD hubs) and gray diamonds (BHD nodes)] are shown for different values of immunization coverage 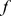, together with the results from immunized bridge nodes identified by CBF (blue circles). Results are obtained by averaging over 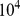 realizations for each value of 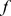. For comparison, the results based on the method proposed by Newman and Girvan [55] are shown as the green dashed lines.

The effectiveness of BHD is further illustrated in Figure 5, which compares the number of weak ties of the bridge hubs and bridge nodes identified for immunization using BHD with that of the bridge nodes identified by CBF. The results show that BHD indeed can identify the nodes with more weak links for removal. In addition, the bridge nodes identified by BHD carry more weak ties than those identified by CBF, further improving the effectiveness. In the Princeton University network, however, the large mean community size (see Table 1) makes it difficult for both BHD and CBF to search for the bridge nodes.

**Figure 6:**
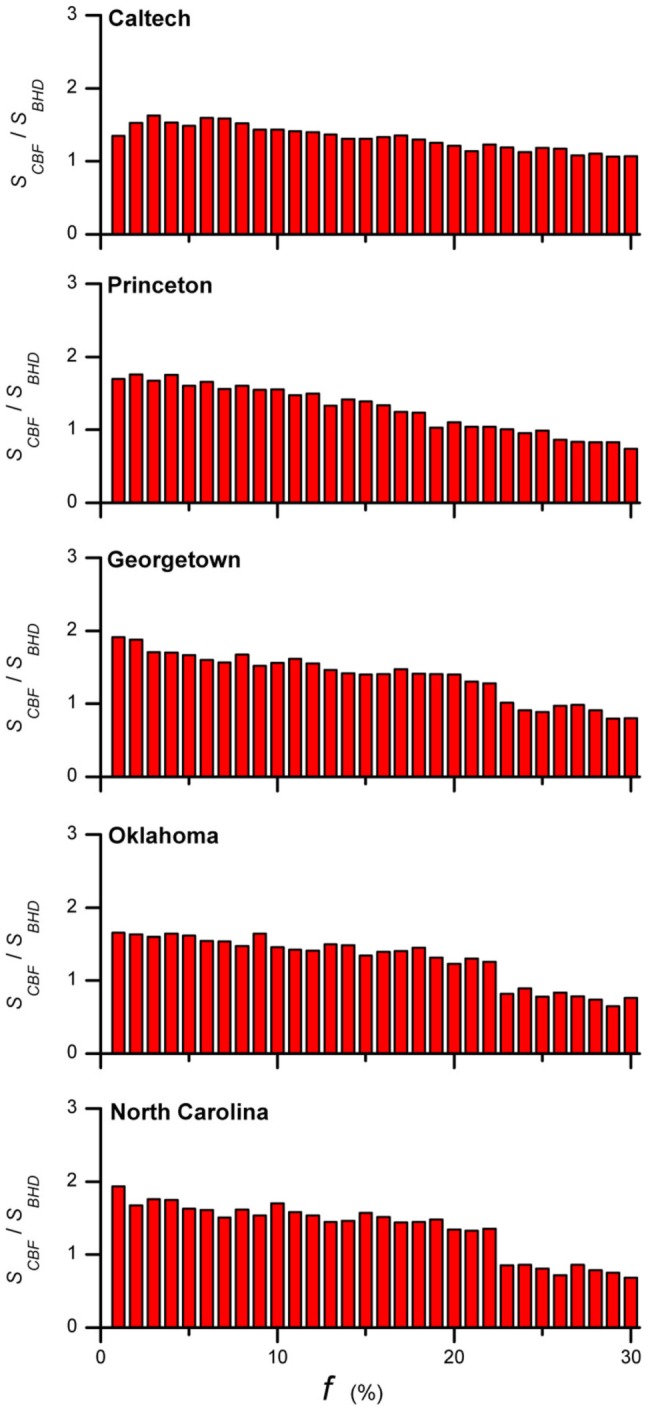
Comparison of number of nodes visited before identifying immunization nodes in CBF and BHD. The ratio 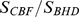 is shown of the number of nodes visited by the self-avoiding walks in CBF and BHD for achieving an immunization coverage 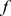. A large 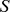 implies a longer search for immunization nodes. The results show that BHD identifies the immunization nodes faster than CBF for 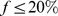. Results are obtained by averaging over 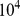 realizations for each value of 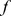.

A more efficient algorithm is one that visits fewer nodes before identifying the targeted nodes for immunization. Let 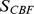 and 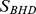 be the numbers of nodes visited by the self-avoiding walks before getting at the targeted nodes. Figure 6 gives 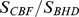 for the five empirical networks. For small immunization coverage (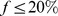), 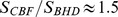, indicating that BHD is a more efficient algorithm.

**Figure 7:**
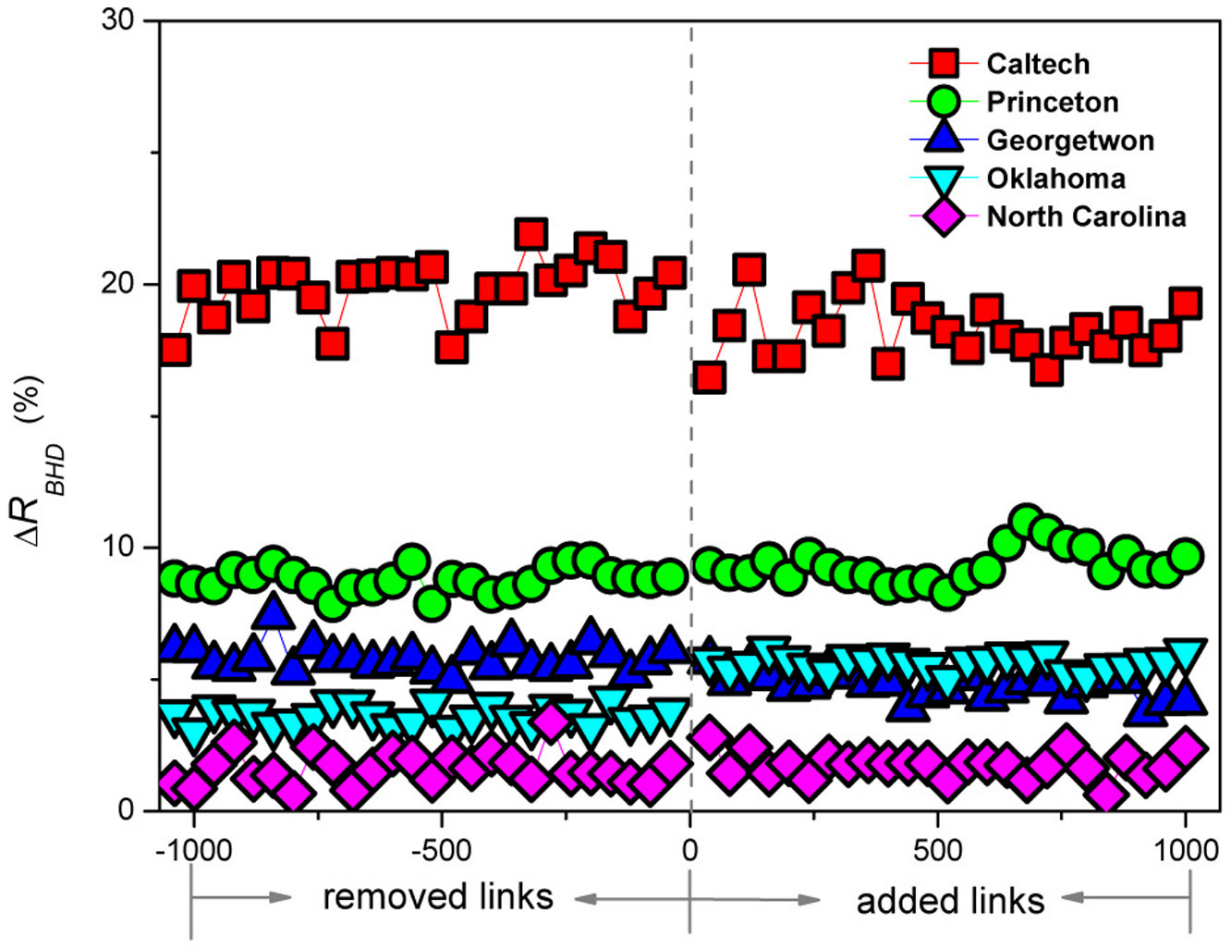
Robustness of BHD in networks with noise as modeled by random addition and removal of links. The quantity 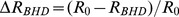 is shown as a function of the number of links randomly added to or removed from an empirical network, where 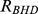 and 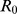 are the final epidemic ratios when BHD is applied and not applied to the modified network, respectively. The robustness of BHD is indicated by the relatively stable values of 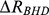. Results are shown for each of the five empirical networks as labeled. The immunization coverage is 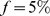. The SIR parameters are 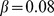 and 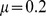. Results are obtained by averaging over 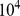 realizations for each value of added and removed links.

Another aspect of an effective algorithm is the robustness to errors or noise in network information. Such errors are common in social networks due to, for example, the inconsistency for two individuals to express their relationship [52]. Using an empirical network, we have tested the robustness of BHD by adding or removing a number of links from the network. SIR dynamics is then studied on the modified network without and with the BHD algorithm. The resulting final epidemic ratios are denoted by 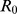 and 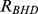, respectively. Figure 7 shows the results of  R_BHD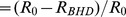 for different numbers of links added or removed, with a constant immunization coverage of 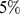. The results show that BHD performs well even when the networks are modified randomly. More results corresponding to other values of immunization coverage are shown in Figure S4.

## Summary and Discussion

The heterogeneous distribution of weak ties in real-world community networks points to the importance of the bridge hubs in the control of transmission of information or diseases but these nodes are difficult to identify. We have proposed and studied the effectiveness of a bridge-hub detector strategy. It is a local strategy searching for bridge nodes and bridge hubs based on the idea of referencing to an expanding friendship circle as a self-avoiding walk proceeds. We applied BHD to simulated networks and empirical networks among students in five US universities constructed by using social network data. In general, BHD is more effective in preventing an epidemic when compared with other local immunization strategies such as ACQ and CBF, for a practical range of immunization coverage. It gives a smaller final epidemic ratio and peak prevalence. Its effectiveness can be attributed to better identification of immunized nodes that carry more weak ties than those picked up by ACQ and CBF. BHD thus breaks up the community networks more effectively and suppresses an epidemic. Although BHD and CBF are both based on self-avoiding walks, BHD is more efficient in that it identifies the immunization nodes after visiting fewer nodes. The good performance of BHD remains robust even when errors exist in the structure of the underlying network.

In general, BHD is capable of identifying bridge nodes with more weak ties and it is particularly useful in dealing with community networks with a broad distribution in the number of weak ties among the bridge nodes. It can be readily generalized to other tasks. For example, mistakenly chosen nodes could be reduced by requiring that a node must have been identified twice or more times before it is immunized. The algorithm can also be applied to overlapping [50] and time-varying [53] community networks.

## Materials and Methods

We have studied the effectiveness of our local immunization strategy in simulated and empirical community networks using the susceptible-infected-recovered (SIR) epidemiological model. Here, we give the details with respect to the following issues: community-network construction, bridge-hub detector algorithm, and SIR dynamics.

### Simulated and empirical community networks

The simulated community networks of different modularity 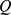 are generated by the algorithm given in Ref. [45]. There are 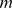 independent random communities. In each community, 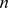 nodes are randomly connected so that the mean degree is 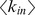. These communities are then connected randomly by 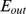 links. The simulated community network thus has a total of 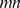 nodes and 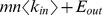 undirected links. We used the same set of parameters as in Ref. [45], namely 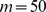, 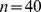, 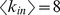, and 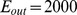. After generating the network, the modularity 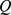 can be evaluated according to the definition given in Ref. [25]. The modularity 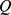 can be varied by rewiring some inter-community links into intra-community links, following the rewiring procedures given in Ref. [45]. While the modularity increases with rewiring, the degree heterogeneity remains nearly unchanged.

Empirical community networks are constructed using the collegiate social network data from Facebook (www.facebook.com) studied in Ref. [27]. The data of five universities in the US are studied (Available: https://code.google.com/p/socialnetworksimulation/). They include data of students from the California Institute of Technology (Caltech), Princeton University, Georgetown University, University of Oklahoma, and University of North Carolina. The data provide information on the dormitory, majoring subject and year of class of the members. Based on the data, a community network is constructed [45] by linking up two members when (i) they are online friends of each other, and (ii) they belong to the same dormitory or the same major in the same year of study. The largest connected component of the network is then retained for carrying out our study. Basic statistical properties of the five empirical networks are given in Table 1. These networks exhibit the small-world character with a high clustering coefficient and a short average path length. The high modularity indicates a strong community feature.

### Local Immunization Strategies and Bridge-Hub Detector

The present paper compares results based on our bridge-hub detector algorithm (BHD) with those obtained by other local strategies based on acquaintance immunization (ACQ) and the community bridge finder (CBF). Before discussing the BHD algorithm, we outline the ideas behind ACQ and CBF.

The ACQ algorithm immunizes randomly chosen acquaintances of randomly chosen nodes [24]. In ACQ, a node is picked randomly and then a neighbor of the chosen node is picked randomly for immunization. The procedure is then repeated until the desired immunization coverage is achieved. Therefore, a node that is a neighbor of many other nodes, i.e., a hub, will have a higher chance to be chosen for immunization. The algorithm does not require any information about the degree of the nodes. It is efficient for large networks with a broad degree distribution. A similar algorithm that requires a node to be chosen two times according to the ACQ random-pick processes (ACQ2) before it is immunized has also been studied [24]. Comparing with ACQ, ACQ2 has a higher probability of immunizing the hubs.

**Figure 8:**
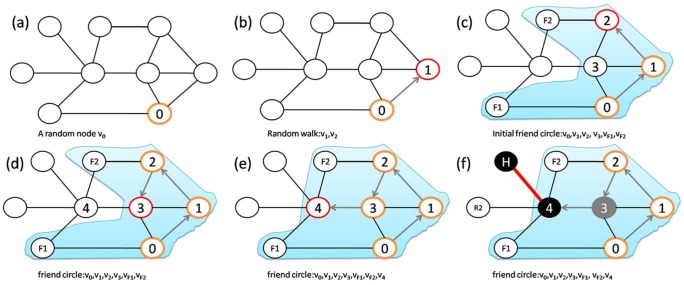
Schematic illustration of CBF strategy and BHD strategy. **The CBF strategy**: (a) A self-avoiding walk starts at a randomly chosen node 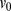. (b)-(c) The walk visited 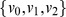 after two steps. The node 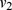 does not have any other links back to 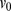 and 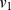 other than the link that took the walk from 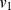 to 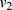. The node 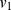 is a potential candidate of a bridge node. Two neighbors of 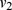, namely 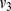 and 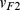, are randomly picked and each is examined for connections to the visited set of nodes. As 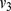 has links with 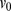 and 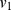, the target node 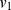 is dismissed as a bridge node and the walk continues. (d) The walk moves to 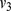 after three steps. The node 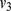 has links to previously visited nodes 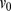 and 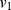, and thus the walk continues. (e) The walk moves to 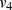 after four steps. The node 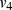 does not have any other links back to previously visited nodes other than the link that took the walk from 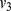 to 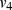. The node 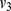 is a potential target of a bridge node. Two neighbors of 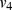 are randomly chosen. (f) If 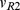 and 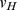 are chosen, these nodes do not connect back to the previously visited nodes and 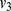 is identified as a bridge node and immunized. **The BHD strategy**: (a) A self-avoiding walk starts at a randomly chosen node 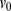. (b)-(c) The walk visited 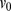, 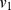 and 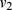 after two steps. The set 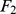 is the union of all the neighboring nodes or friendship circles of 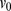, 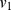 and 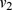, as shaded in (c). (d) The walk moves to 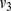 after three steps. The friendship circle 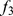 of node 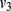 consists of 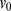, 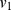, 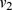, and 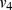. As all the nodes in 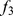 either belong to 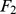 or have a link to at least a node in 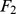, the node 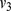 is not a potential target for immunization. (e) The union of friendship circles is updated to 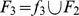 as shaded. The walk continues and reaches node 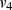 after four steps. The friendship circle 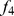 of 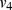 consists of 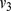, 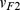, 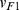, 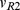 and 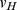, among them 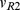 and 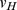 do not belong to 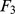 and do not have a link to nodes in 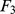. The node 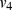 is then identified as a bridge node for immunization. In addition, among those nodes in 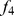 that cannot be linked back to 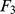, one node, e.g., 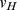, is randomly chosen and identified as a bridge hub for immunization.

**Figure 9:**
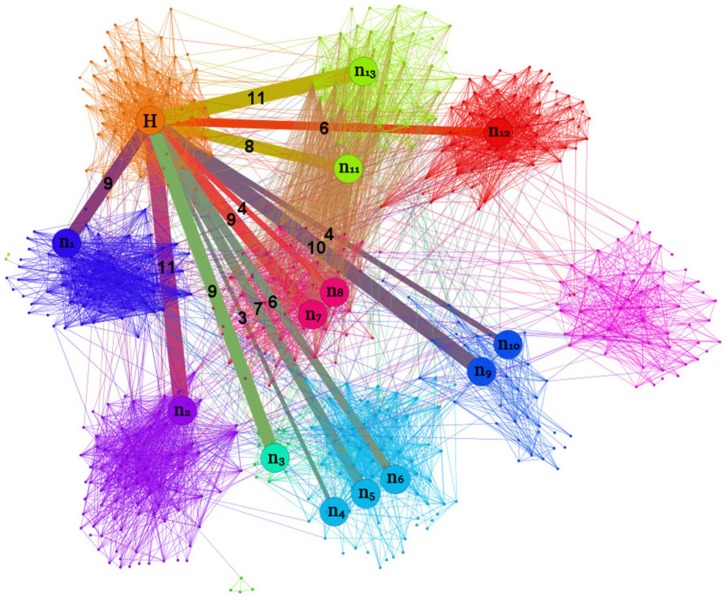
Visualizing the community structure and a bridge hub in the Caltech network. The Caltech network can be divided into 13 communities based on the method of Newman and Girvan [55], as illustrated by the different colors. Also illustrated is a bridge hub 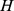 that carries the largest number of weak ties and connects with other bridge nodes labeled as 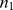 to 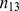. The number on a weak tie shows the difference in the numbers of weak ties originated from the node 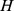 and the bridge node at the other end of the weak tie.

Bridge nodes are important for transmission through community networks, but ACQ does not aim at the bridge nodes. In contrast, CBF is designed to search for bridge nodes [45]. It is an algorithm based on self-avoiding walks. To identify a bridge and its associated bridge node for immunization, a self-avoiding walk is executed starting from a randomly chosen node 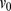. The following procedure is taken after every step when the walk has visited three or more nodes (see Figure 8). For a walk after 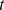 steps (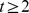), the set of all nodes visited in the 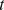 steps so far is denoted by 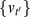 for all 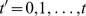. Thus, 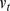 is the node that the walk currently locates after step 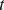 and 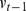 is the node visited at step 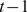. The first check is to examine whether the node 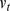 has a link or several links to the nodes in the set 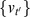 other than the link between 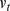 and 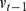. If it is the case, the self-avoiding walk continues to step 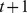. If not, then 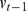 is considered a possible target of a bridge node. To determine whether the node 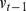 is a bridge node, two nodes are randomly chosen among all the possible nodes that the walk could go in step 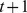, i.e., two neighbors of the node 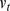 are randomly chosen from all the neighbors (except the node 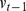 due to the self-avoiding restriction of the walk). In the case that there is only one neighbor to choose from, the only neighbor will be considered. If there exists a path from any of the two chosen nodes back to any node in the set 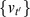, then the node 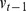 will not be regarded as a bridge node and the walk moves on from 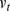 to some node 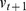. If there exists no path back to the set 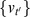 from both of the chosen nodes, 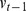 is regarded as a bridge node that connects two communities and it will be immunized. A new self-avoiding walk will start again at another randomly chosen node and the procedure is repeated until the desired immunization ratio is achieved. The idea behind CBF is that a community is formed by a circle of close friends, and thus when the two randomly chosen neighbors of 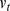 cannot be traced back to the community that 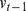 belongs to, the link between 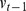 and 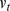 is likely to be a bridge between two communities and so the node 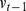 is a bridge node. In practice, two additional checks are implemented to shorten the computing time [45]. Firstly, the number of nodes registered in a running path is kept at the length of ten, using the latest ten nodes visited. Secondly, the number of visits by any random walk for each node is recorded. When the number 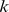 of visits equals a certain number (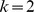 in Ref. [45]), the node is immunized. We have implemented CBF following the algorithm in Ref. [45], where it has been shown that, without prior knowledge of the community structure, CBF is more efficient than ACQ and other immunization strategies that target at the different kinds of hubs. In its design, CBF does not aim at identifying the bridge hubs. In real-world community networks such as those in Facebook [47], worldwide air traffic network [48], and the five collegiate community networks studied here, the heterogeneous distribution in the number of weak ties among bridge nodes indicates the importance of bridge hubs. Figure 9 shows an example of a bridge hub through a visualization of the students' network in Caltech.

We propose a bridge-hub detector (BHD) for more effective immunization in community networks. It is a local algorithm based on an expanding friendship circle and it does not rely on prior knowledge of the community structure. As in CBF, the algorithm starts with a self-avoiding walk at a randomly chosen node. The following procedure is taken after every step when the walk has visited three or more sites (see Figure 8). Let 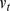 be the node that the walker locates after step 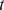 and 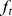 be the set of all neighbors of the node 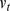. For a walk up to 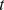 steps (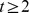), the following steps are carried out between the set 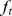 and the set 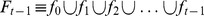. The set 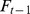 is the union of all the friendship circles of all the nodes visited by the walker up to the step 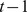 and it expands as the self-avoiding walk proceeds. Apparently, we have 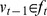. If every other node in 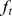 either already belongs to the set 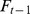 or has at least a link to the nodes within 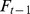, 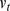 will not be taken as the target of immunization and 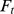 will be updated to 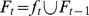. The self-avoiding walk then moves on from 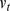. Otherwise, there must be at least a node in 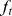 that is not a member in 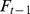 and is not connected to the nodes in 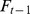. The node 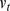 is then targeted for immunization. In addition, among the nodes in 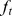 that do not belong to 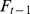 and cannot be linked back to 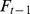, one node (call it 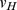) is randomly chosen for immunization. A pair of nodes, 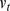 and 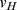, are immunized and the self-avoiding walk stops. A new walk is initiated at another randomly chosen node again. The procedure is repeated until the desired immunization ratio is achieved. Similar to the idea in ACQ, the immunized node 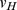 in every self-avoiding walk is likely to be a bridge hub in the presence of heterogeneous distribution of weak ties among the bridge nodes, while 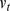 is a bridge node. The algorithm is illustrated schematically in Figure 8. Practically, we terminate a walk when it fails to identify immunization nodes after 20 steps. As in CBF, BHD is effective when the search for immunization nodes begins after 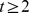 steps. Figure S5 in Supporting Information gives results when the search begins after different numbers of steps. Through computational complexity analysis (see Text S1 in Supporting Information for more details), the algorithms ACQ, CBF and BHD take on the worst-case run times that go as 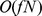, 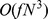 and 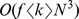, respectively. For the different algorithms working on systems with the same parameters (the same network size 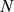 and immunization ratio 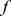 etc.), ACQ is the fastest, followed by CBF and BHD is the slowest. On a sparse network with small 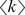, BHD will have almost the same computational complexity as CBF.

### Epidemic Dynamics

The susceptible-infected-recovered (SIR) epidemiological model [54] is used to test the performance of different immunization strategies on community networks. In the model, each node in the network represents an individual that can be in one of three states: susceptible (S), infected (I), or refractory/recovered (R); and each link represents a connection that could spread a disease. When an immunization strategy is executed, a percentage of nodes are first removed from the network by implementing ACQ, CBF or BHD. The remaining nodes are set to the susceptible state. To initiate an infection, a node is randomly chosen and turned into the I state. In a time step, a susceptible node will be infected with a probability 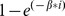, where *i* is the number of infected nodes among the connected neighbors of the node. Here, 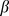 is a parameter characterizing the transmission rate. Meanwhile, an infected node has a recovery probability 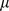 to turn into the R state. Once an individual is in the R state, there will be no further change. In the simulations, the parameters are taken to be 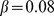 and 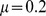 to ensure an outbreak and the state of every node is updated synchronously in every time step. The dynamics ends when all infected nodes have recovered. The resulting population contains nodes only in the S state and R state. We record the *final epidemic ratio* 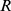 corresponding to the density of R nodes at the end of the epidemic, the *peak prevalence* 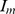 corresponding to the highest density of infected nodes in the network during the epidemic, and the *duration of epidemic* before the dynamics ends, and compare these quantities for ACQ, CBF and BHD.

## Supporting Information

Figure S1
**Comparison of peak prevalence of immunization algorithms in empirical networks.** The differences in the peak prevalence (left panel) 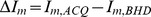 between ACQ and BHD, and (right panel) 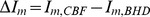 between CBF and BHD are shown for each of the five empirical networks as a function of the immunization coverage 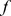. A positive value indicates that BHD is more effective than the other algorithms. The comparison is similar in features with those shown in Figure 3 for the differences in the final epidemic ratios. Results are obtained by averaging over 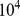 realizations for each value of 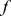.(EPS)

Figure S2
**Comparison of residual mean degrees of different immunization algorithms in empirical networks.** The differences in the mean degrees 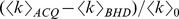 (left panel) and 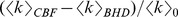 (right panel) using different immunization algorithms are shown for each of the five empirical networks as a function of the immunization coverage 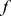. Here, 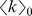 is the mean degree of the network before an immunization algorithm is applied. A positive value indicates that BHD is more effective in reducing the mean degree and thus breaking up the network. Results are obtained by averaging over 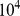 realizations for each value of 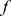.(EPS)

Figure S3
**Comparison of duration of epidemic of different immunization algorithms in empirical networks.** The differences in the duration of epidemic between ACQ and BHD (left panel) and between CBF and BHD (right panel) are shown for each of the five empirical networks as a function of the immunization coverage 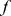. Results are obtained by averaging over 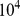 realizations for each value of 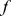.(EPS)

Figure S4
**Robustness of BHD in networks with noise as modeled by addition and removal of links for** 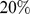 **immunization coverage.** As in Figure 7, the quantity 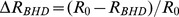 is shown as a function of the number of links randomly added to or removed from an empirical network. Results are shown for each of the five empirical networks as labeled. The immunization coverage is 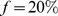. The SIR parameters are 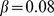 and 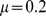. Results are obtained by averaging over 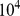 realizations for each value of added and removed links.(EPS)

Figure S5 **Effects of starting the search for immunization nodes from different number of steps on the final epidemic ratio.** Results in the main text and figures are obtained by starting the search for immunization nodes two steps after a self-avoiding walk began. Here, results for starting the search after 1, 2, 3, 4, 5 steps are shown for each of the five empirical networks.
(EPS)

Text S1 **Computational Complexity Analysis.**
(PDF)
