# Supplementary material for: An Efficient Immunization Strategy for Community Networks
Source: PLoS One. 2013 Dec 20;8(12):e83489. doi: 10.1371/journal.pone.0083489 (PMC3869806; doi:10.1371/journal.pone.0083489)
Supplement: Text S1 — Computational Complexity Analysis. (PDF) [file pone.0083489.s006.pdf]

**Text. S1. Computational Complexity Analysis.** The BHD strategy falls into the general category of immunization algorithms based on the self-avoiding walks [1]. Starting with a self-avoiding walk at a randomly chosen node, we examine the overlap and the existence of links from $f_t$ (i.e., all the neighbors of the last node after step $t$) back to $\mathcal{F}_{t-1}$ (i.e., the union of the friendship circles of all the visited nodes up to step $t-1$) in the trail of the walk, and this examination procedure is taken after every step once the walk has visited three or more sites.

For a randomly chosen node, it takes time $O(1)$. A self-avoiding walk takes the worst-case time $O(NlnN)$ [2], where $N$ is network size. Following the above examination procedure, the connection relations between each node in $f_t$ and all the nodes in $\mathcal{F}_{t-1}$ are examined after each step of the walk, which takes the worst-case time $O(\langle k\rangle N)$, where $\langle k\rangle$ is the average degree of the network. As $N$ nodes may be visited at most by the walker, the whole process of this examination takes the worst-case time $O(\langle k\rangle N^2)$. When a walk stops, one bridge node and one bridge hub are identified. Thus, an identification process based on the self-avoiding walks takes the worst-case time $O(1 + NlnN + \langle k\rangle N^2)$=$O(\langle k\rangle N^2)$. To achieve the desired immunization ratio $f$, the entire algorithm runs in time $O(f\langle k\rangle N^3)$. Analogously, the ACQ and CBF take the worst-case run times that go like $O(fN)$ [3] and $O(fN^3)$, respectively.

1. Salathe M, Jones JH (2010) Dynamics and Control of Diseases in Networks with Community Structure. PLoS Comput Biol 6:1000736
2. Turban L (1983) Generalised self-avoiding walk. Physics A, 16: L643.
3. Chao G, Jiming L, Ning Z (2011) Network Immunization with Distributed Autonomy-Oriented Entities. IEEE Transactions on Parallel and Distributed Systems 22: 1222-1229.
